# Endodontic Treatment of Maxillary Premolar With Three Roots: A Case Report

**DOI:** 10.1155/2024/5525349

**Published:** 2024-07-22

**Authors:** Chen Jiannan, Zhao Yangpeng, Sun Huanhuan, Zhu Qiang

**Affiliations:** The First Affiliated Hospital of Naval Medical University, No. 168 Changhai Road, Yangpu 200433, Shanghai, China

**Keywords:** anatomical variation, first premolars, pathological report, root canal abnormalities, root canal therapy, three root canals

## Abstract

**Background:** The aim of this case was to understand and treat the intricate root canal anatomy observed in complex maxillary first premolars, particularly those exhibiting three root canals, emphasizing the significance of understanding root canal morphological variations in their treatment.

**Conclusions:** This case reported the methods of treating three-root canal maxillary first premolars, including warm vertical compression technique, root canal microsurgery, and the application of rubber dam. The utilization of dental operating microscopes, various surgical strategies, and key assessments of X-rays and cone beam computed tomography (CBCT) scans were all essential steps for achieving accurate and safe root canal therapy.

## 1. Introduction

This case report was prepared according to the PRICE 2020 Guidelines [1].

### 1.1. Method

A 23-year-old male presented with left upper posterior dental, experiencing pain due to food impaction for more than 3 weeks, was diagnosed with chronic apical periodontitis of maxillary left first premolar. Three root canals, including proximal buccal, distal buccal, and palatal roots, were prepared using the ProTaper rotation system. One week later, the third root canal was filled in the oral cavity. Cone beam computed tomography (CBCT) confirmed that the upper premolars of the three canals appeared symmetrically. After the completion of root canal filling, the CBCT examination was conducted, and three-dimensional root canal images were reconstructed. After 3 months of observation, the teeth were repaired with fiber post and all-ceramic crown. No abnormalities were found during the follow-up examination after 6 months.

## 2. Case Presentation

Maxillary first premolars present significant challenges in pulp treatment due to their variable root number, complex root canal structure, diverse root orientation and longitudinal depression, and varying pulp cavity configurations. The variation in maxillary first premolars is significant, with the majority exhibiting two root canals, while the incidence of three root canals ranges from 0.5% to 7.5% [2–4]. Determining the number of root canals and the location of root canal orifices in the early stage of treatment is crucial for the success of root canal therapy.

According to domestic and foreign literature reports, the global prevalence of multiple root canals in maxillary first premolars was 93.5%, with the incidence of three root canals being 1% [5]. The variation of the root canal system may be associated with race and gender [2, 6]. Analysis of geographical areas showed that the prevalence of premolars in European and African countries was higher compared to that in Asian regions [5]. Current research results from China showed that the incidence of multiple roots in maxillary premolars was 16.8%−37.7% [5–8]. Similarly, the incidence of this morphological feature is 51.2%−96.6% in various countries in Europe [5, 7–12]. The root morphology of the maxillary first premolars showed the incidence of three root canals in 0.4%, 2.5%, and 2.7% in Asia, Europe, and Oceania, respectively, and 1.87% in the Han Chinese population. Gender studies showed that root canal variation was 96.2% in men and 92.6% in women [5], and prevalence was 2.4% in men and 0.9% in women. Previous case reports show that three maxillary premolars have several root canals and root structures: three roots in one root, two in buccal roots, one in palatal roots, three separate roots and canal [13–15], and a rare four separate canal [16–18] in three premolars. This case report is aimed at reporting a rare case of maxillary first premolar three canals and their root canal therapy.

## 3. Report

The patient was a 23-year-old male. On November 23, 2022, he presented with occasional intermittent spontaneous pain due to caries in the left upper posterior tooth.

Clinical Examination

The distal adjacent surface of the left maxillary first premolar exhibited resin filling, with visible edges and loose filling material upon probing. Pain was elicited upon exploration, with slight percussion tenderness noted. No evidence of loosening or redness and swelling of the gingiva was observed. The temperature test was sensitive. CBCT was taken with consent by the patient himself.

### 3.1. CBCT Film (Figures 1(A), 1(B), and 1(C))

The CBCT revealed deep caries and pulp cavity, with a low-density image observed at the root tip and widening of the periodontal ligament.

### 3.2. Diagnosis

#### 3.2.1. Chronic Apical Periodontitis of the Left Upper First Premolar

**Figure 1:**
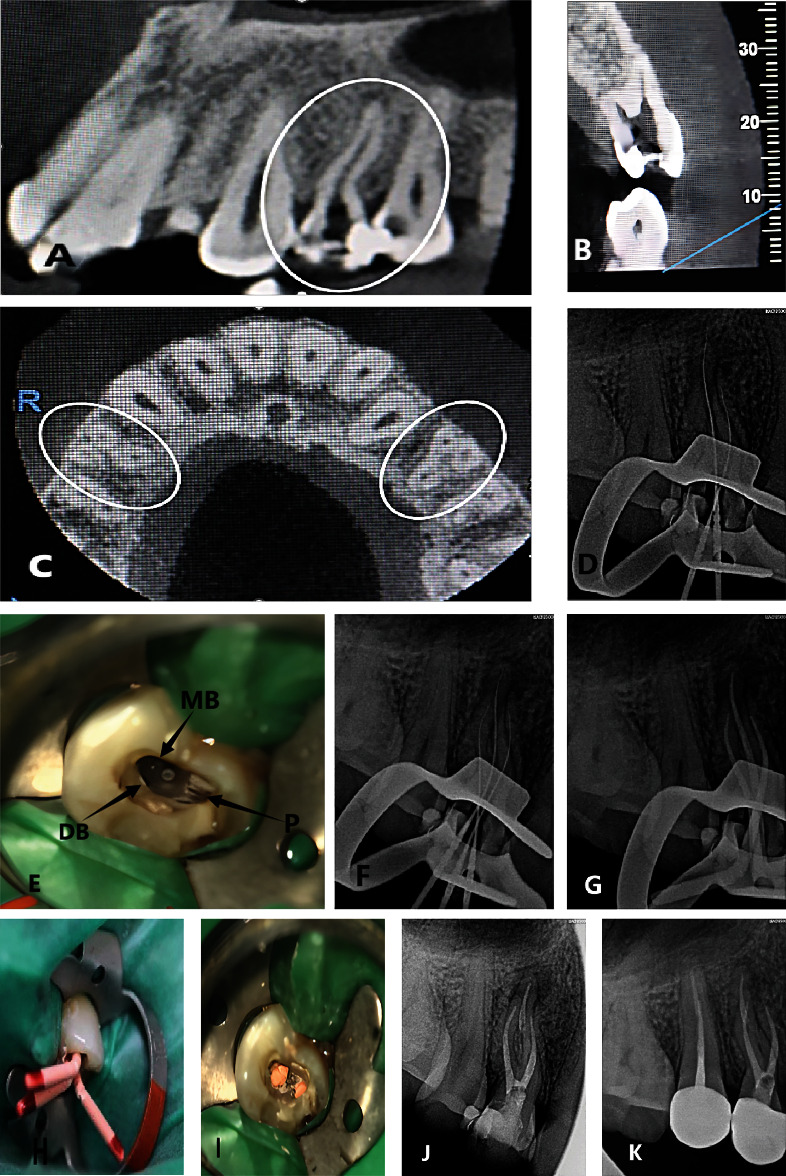
The treatment process of maxillary left first premolar tooth ((A–C) preoperative, (D–I) intraoperative, (J, K) postoperative). (A–C) CBCT images represent 3 root configurations, showing apical section from left to right and two sagittal screenings of buccal and lingual root of maxillary left first premolar teeth; (D) diagnostic periapical radiograph; (E) root canal orifice photograph under microscope; (F) 3 root canal diagnostic periapical radiograph; (G) intraoperative tip film; (H) root canal test tip under microscope; (I) root filling film; (J) palatal root postfilm; (K) periapical radiograph 6 months after full crown restoration.

Treatment, discovery, and confirmation of three root canals: Root canal therapy of left upper first premolars. Informed consent was obtained from the patient. Epinephrine-containing lidocaine local infiltration anesthesia for dental procedures, with the rubber septum. In the maxillary left first premolar, the pulp was accessed following pulp exposure, and the mesiobuccal root canal was detected using the DG-16 probe. One root canal was identified on the palatal side. The root canals were unobstructed with 10 stainless steel K (MANI Company, Japan). Two root canals were found to be unobstructed and curved at an angle. Additionally, two pulp extraction needles were removed during the procedure. The length of the root canal was measured using the root canal length measuring instrument (Root ZX, Morita, Japan), with the palatal root canal measuring 21 mm and the buccal root canal also measuring 21 mm. Furthermore, diagnostic wire images were photographed for another maxillary left first premolar (Figure 1(D)). Similar images were observed in the distal buccal root canals, raising suspicion regarding the presence of additional distal buccal root canals. When the dentin canals of the isthmus were repaired under the microscope, two root canals, namely, the proximal buccal and distal buccal root canals, were observed a few millimeters away from the original buccal root canal (Figure 1(E)). The distal buccal root canal was unobstructed, and its length was measured to be 21 mm using the root canal measuring instrument. In order to determine the shape of the root canal, a diagnostic X-ray film (Figure 1(F)) was inserted to reveal the presence of three root canals. Three canals were prepared using ProTaper Niti rotary instruments (Den Berg, USA) to F2, and the root canals were irrigated with 5.25% sodium hypochlorite solution. After the operation, the drug was blocked by camphor phenol in the root canal, and a follow-up appointment was scheduled for one week later. To further confirm the findings, we extracted a CBCT of the maxillary left first premolar， showing the screenshots of the apical section and the sagittal plane of the mesiobuccal and distal roots of maxillary left first premolar teeth (Figure 1(C)), clearly illustrating the presence of 3 roots in maxillary left first premolar teeth. Additionally, it was found that maxillary right first premolar teeth also exhibited 3 roots. A Hplus and F2 06 taper gutta-percha tip were used. Root canal filling was performed using vertical compression of warm vertical. The postoperative X-ray showed that all the 3 canals were filled (Figures 1(G) and 1(H)). After filling, the surgical microscope revealed two root canal orifices on the buccal side and one on the palatal side (Figure 1(I)). On March 22, 2023, the patient was revisited with no discomfort symptoms. Maxillary left first premolar teeth were required to be restored with a full crown. After the clinical examination by the restorative dentist, no tooth discomfort was found, and postcrown restoration was carried out(Figure 1(J)). The reexamination on October 27, 2023, showed that 6 months after the restoration with all-ceramic crowns on maxillary left first premolar teeth (Figure 1(K)), the apical bone mineral density was good, with no obvious low-density image, and the outcome of the root canal therapy was ideal.

## 4. Discussion

The variability of maxillary first premolars is considerable [3]. According to the Vertucci classification, the method is divided into 8 categories [19]. The details are as follows: 1, 2-1, 1-2-1, 2-2, 1-2, 2-1-2, 1-2-1-2, 3 root canals. This case is Type VIII, indicating 3 obvious root canals extending from the pulp chamber to the apical (i.e., 3 root canals).

Root canal therapy of maxillary premolars with three canals is challenging, and it is easy to fail due to the omission of root canals. The success of this case can be attributed to the timely use of X-ray film and CBCT to accurately locate the three root canals and complete the treatment.

A total of 27.3% of the maxillary first premolars exhibited bilateral symmetry [4]. After performing CBCT in this case, it was found that the healthy side tooth maxillary right first premolar also had three canals. Therefore, attention should be paid to the symmetry of the three canals in each individual. CBCT imaging has several advantages, including high resolution, flexibility, convenience, accuracy, and preservation of tooth structure without damage [20]. Moreover, CBCT imaging provides measurement data that closely resemble real dimensions, enabling its wide application in tooth-related measurements and analyses. Current reports show that CBCT is an effective tool for the clinical diagnosis of complex and variant root canals. Preoperative CBCT examination plays a crucial role in assisting the identification of variant root canals and locating root canal orifices [21]. CBCT provides a fundamental anatomical basis for understanding the intricate root canal morphology of maxillary premolars.

This case was treated with a rubber stamp and a microscope. The rubber dam helped maintain a clear field of view during the treatment. In dental treatment, a clear field of view is crucial for clinicians, as it prevents saliva, blood, and medication from obstructing vision, thus improving the efficiency and accuracy of treatment [22]. The morphology of the pulp floor can be observed under the microscope, which helped reduce the likelihood of missing root canals [23–25]. In this case, the dentine shoulder collar of the distal buccal root canal orifice was accurately removed under the microscope, facilitating the establishment of a straight path for root canal clearance. Compared with traditional root canal therapy, the widely popularized microroot canal therapy is particularly significant in terms of both curative effect and root canal detection rate. In this case, the ProTaper Nickel titanium system was used to treat the root canal and repeatedly washed with 3% hydrogen peroxide and normal saline to completely remove the stain layer. According to the case analysis, the same as most scholars [26–28], the use of a nickel–titanium system can reduce the operation time, preserve the original shape of the root canal, and reduce the complications after root canal treatment, and the success rate of root canal treatment over time is more than 96%.

The case report design, while valuable for highlighting unique clinical scenarios, has inherent limitations. Our study is limited by the small sample size, which restricts the generalizability of the findings. Additionally, the lack of a control group or comparative analysis with alternative treatment modalities may affect the interpretation of the results. Future research involving larger cohorts and controlled studies would provide a more comprehensive understanding of the treatment approaches for complex endodontic cases.

## 5. Conclusion

Combined with this case, we can understand that X-ray films before treatment and auxiliary CBCT are helpful in fully grasping the shape of variant root canals, contributing to the success of curved root canal therapy. Although most studies have shown that the maxillary first premolar typically has two root canals, cautious clinicians should always approach root canal therapy with an open mind and anticipate anatomical variation [29–31]. Therefore, it is recommended to utilize advanced tools such as CBCT and microscope when dealing with teeth exhibiting complex root canal anatomy. This approach ensures the accuracy required to manage complex cases and improves the ultimate success rate of root canal therapy. Good visual enhancement and accurate imaging interpretation of the dental pulp cavity are crucial for the success of root canal therapy. This case further enriches the understanding of the anatomical variations of the maxillary first premolar and serves as a valuable reference for other similar cases in the future.

## Data Availability

The authors have nothing to report.
